# Notes and News

**Published:** 1904-05-07

**Authors:** 


### Notes and News.

The annual meeting of the Chelsea Hospital will be held on May 17 under the presidency of Lord Glenesk.

The late Mr. Judah Varicas has bequeathed £2,500 to the Home and Hospital for Jewish Incurables.

Mrs. A. H. Lewis has forwarded £2,500, the first quarterly payment of her annual subscription, to King Edward's Hospital Fund.

Dr. Rose Bradford will deliver the Croonian Lectures on June 7th, 9th, 14th, and 16th, taking for his subject "Bright's Disease and its Varieties."

The late Mr. William Martin Bickerstaff, of Regent's Park, has bequeathed £1,000 to the Home for Incurables, Putney, and £1,000 to St. Bartholomew's Hospital, and £550 to its Samaritan Fund.

The Director of Public Health and Charities in Philadelphia has established a fresh-air retreat for sick and needy children. It is a private undertaking and the patients will be sent by the director and various societies.

The Cannock Rural District Council have opened a new Isolation Hospital. It consists of three blocks, two being ward blocks connected by a covered way. The accommodation is for 16 patients, and the cost was about £2,000.

We commented last week upon the want of faith in the efficacy of a residence in an inebriate home to effect a cure for drunkenness which was exhibited in court by judge and missioner last week. An amusing testimony to this opinion comes this week from a woman convicted of drunkenness at another court, and recommended for a home. When informed of this decision the lady—who had on a previous occasion resided in a home and found it "very comfortable"—remarked, "Well, my lord, it will simply be wasting money."

The last report on the quality of the London water supply was very satisfactory.

The Egyptian Government has invited Professor Koch, who is returning from South Africa, to spend a few days in Egypt to study the cattle plague now prevalent, and to suggest a remedy.

A systematic daily medical inspection of elementary schools has been established in Philadelphia. The parents of children who need medical attention will be communicated with and the decision as to the method of securing medical treatment will be left to them.

The first convalescent home for New York is to be established at New Dorp, Staten Island, by the St. John's Guild. Three hundred and twenty patients will be accommodated at first and it is intended to extend the system as quickly as possible. Dr. William Maynard came to London to study the system of convalescent aid, which has hitherto been nearly entirely neglected in New York.

The Cork Branch of the Association for the Prevention of Consumption has obtained the sanction of the Local Government Board for the establishment of a sanatorium. The Cork Association has already £5,000 guaranteed for the purpose. Mr. Richard Barter, of St. Anne's Hill, Cork, has offered a site of 20 acres, or a sum of £500 towards the purchase of another site if the one he offers is not deemed suitable.

A writer in the *Times*, who signs himself Colonist, sends a strong defence for the Natal medical officers, whose services during the South African war were ignored during the war Commission Inquiry. He states that Natal sent 13 volunteer medical men into the field, and that the only Itombi hospital which escaped criticism from the Court of Inquiry, appointed by Sir George White, was that under the control of Natal medical officers.

An interesting discussion took place at a meeting of the managers of the Metropolitan Asylum District last week on a proposal to allow the medical superintendent of the Joyce Green Small-pox Hospital, in conjunction with Dr. Martin, director of the Lister Institute, to carry on experimental investigations in the causation of small-pox. The proposal was adopted, and instructions given that arrangements should be made to secure the licence necessary to permit of inoculations being made at the hospital.

Three prosecutions have recently been made against the sellers of adulterated cod liver oil. It was stated that the value of cod liver oil was 15s. 2d. per gallon, and of fish oil, the adulterant used, the value was 2s. 2d. Further, it was declared that the use of fish oil was detrimental. All three sellers were convicted of selling the adulterated oil, though the evidence was not quite conclusive that in all three cases the retailer was responsible for the adulteration. In only one case had the wholesale vendors issued a warranty with the oil.

